# Efficacy and Safety of Dihydroartemisinin-Piperaquine for Treatment of *Plasmodium vivax* Malaria in Endemic Countries: Meta-Analysis of Randomized Controlled Studies

**DOI:** 10.1371/journal.pone.0078819

**Published:** 2013-12-03

**Authors:** Cho Naing, Vanessa Racloz, Maxine Anne Whittaker, Kyan Aung, Simon Andrew Reid, Joon Wah Mak, Marcel Tanner

**Affiliations:** 1 School of Population Health, University of Queensland, Herston, Australia; 2 International Medical University, Kuala Lumpur, Malaysia; 3 Swiss Tropical and Public Health Institute, Basel, Switzerland; National Institute of Medical Research, United Kingdom

## Abstract

**Background:**

This study aimed to synthesize available evidence on the efficacy of dihydroartemisinin-piperaquine (DHP) in treating uncomplicated *Plasmodium vivax* malaria in people living in endemic countries.

**Methodology and Principal Findings:**

This is a meta-analysis of randomized controlled trials (RCT). We searched relevant studies in electronic databases up to May 2013. RCTs comparing efficacy of (DHP) with other artemisinin-based combination therapy (ACT), non-ACT or placebo were selected. The primary endpoint was efficacy expressed as PCR-corrected parasitological failure. Efficacy was pooled by hazard ratio (HR) and 95% CI, if studies reported time-to-event outcomes by the Kaplan-Meier method or data available for calculation of HR Nine RCTs with 14 datasets were included in the quantitative analysis. Overall, most of the studies were of high quality. Only a few studies compared with the same antimalarial drugs and reported the outcomes of the same follow-up duration, which created some difficulties in pooling of outcome data. We found the superiority of DHP over chloroquine (CQ) (at day > 42-63, HR:2.33, 95% CI:1.86-2.93, *I* ^2^: 0%) or artemether-lumefentrine (AL) (at day 42, HR:2.07, 95% CI:1.38-3.09, *I* ^2^: 39%). On the basis of GRADE criteria, further research is likely to have an important impact on our confidence in the estimate of effect and may change the estimate.

**Discussion/Conclusion:**

Findings document that DHP is more efficacious than CQ and AL in treating uncomplicated *P. vivax* malaria. The better safety profile of DHP and the once-daily dosage improves adherence, and its fixed co-formulation ensures that both drugs (dihydroartemisinin and piperaquine) are taken together. However, DHP is not active against the hypnozoite stage of *P. vivax*. DHP has the potential to become an alternative antimalarial drug for the treatment uncomplicated *P. vivax* malaria. This should be substantiated by future RCTs with other ACTs. Additional work is required to establish how best to combine this treatment with appropriate antirelapse therapy (primaquine or other drugs under development).

## Introduction

According to a recent estimate, *Plasmodium vivax* accounts for up to 50% of malaria cases with prevalence rates between 1% and 6% of the population in South and South East Asia, where the majority of *P. vivax* malaria occurs. In Central and South America and Eastern and Southern Africa, it accounts for 71-81% and 10% of malaria cases, respectively [1–3]. Although *P. vivax* malaria has a reputation of being a benign infection, severe and fatal complications also occur [4] such as maternal anaemia in pregnancy and significant reduction in mean birthweight [5]. Treatment failure due to resistance to chloroquine (CQ) in *P. vivax* was first documented in 1989 among Australians repatriated from Papua New Guinea [3]. Since then, sporadic resistance to CQ has been reported from other countries including Brazil [6], Ethiopia [7], Myanmar [8] and Turkey [9], among others. As such, the recent documented emergence of resistance to CQ in vivax malaria deserves paying attention to *P. vivax* drug sensitivity. Early diagnosis followed by prompt and effective treatment remains a cornerstone for the reduction of malaria-related morbidity and mortality [10]. Along this thread, alternative antimalarial treatments for *P. vivax* are needed [11]. In the development of new antimalarials, dihydroartemisinin-piperaquin (DHP), a newer co-formulated artemisinin-based combination therapy (ACT) consisting dihydroartemisinin (DHA) and (bisquinoline) piperaquine (PPQ), could be considered as an alternative choice. DHP has recently been added to the list of ACT options recommended for the treatment of uncomplicated *P. falciparum* malaria [12]. The artemisinin (ART) component in DHP (i.e. DHA) concentrations peak within 25 minutes post-dose, and DHA is eliminated with a half-life of 30-60 minutes, significantly shortening the period of exposure of a new infection to a single drug [13]. The ability of the relatively potent, short-acting ART derivatives (DHA in our case) to rapidly reduce the parasite biomass [13,14] results in fewer parasites having to be cleared by the longer-acting but intrinsically less active partner drug (PPQ in our case) [15,16]. This subsequently reduces the pool of parasites from which resistance can emerge [13,14,17].

A previous review of 14 trials solely from the Asian region has reported that DHP is safe and highly effective for treatment of uncomplicated *falciparum* malaria [18]. A Cochrane review [19] assessing ACTs, including DHP, for treating uncomplicated malaria was also available. A meta-analysis of DHP was performed to compare efficacy and safety in treating *P. falciparum* per se [20]. Since the publication of these reviews mainly on uncomplicated *falciparum* malaria, there has been a surge of published RCTs undertaken in endemic countries to compare DHP with other antimalarial agents for the treatment of vivax malaria. As the epidemiology of malaria is complex and heterogeneous, with variations over small areas [21] as well as being age dependent [22], information from RCTs across geographic regions and all age groups is valuable.

Taken as a whole, the objective of the present review was to synthesize the available evidence assessing the efficacy of DHP in treating uncomplicated *P. vivax* malaria in people living in malaria-endemic countries.

## Materials and Methods

### Search strategy and selection criteria

We conducted a literature search in MEDLINE, EMBASE, CINHAL, the Cochrane Library and the database of abstract of Reviews and Effectiveness from January 1989 to May 2013. For ongoing and unpublished trials, we also looked at the websites such as http://www.clinicaltrials.gov, http://www.controlled-trials.com, andhttp://www.nci.nih.gov/clinicaltrials. Furthermore, we manually searched the reference sections of the selected studies and relevant reviews to look for any additional studies which were not found in the initial search. Searches were limited to English language and those with human participants. The search terms we used were malaria, *vivax*, treatment, dihydroartemisinin, piperaquine, dihydroartemisinin-piperaquine, Arteken, efficacy, treatment success, treatment failure, safety, tolerability, resistance. We determined the inclusion criteria following the PICO format; (1) Participants (P): those having confirmed *P. vivax* (either microscopy or point-of care rapid-onsite diagnostic test for malaria) mono-infection at enrollment, regardless of age and pregnancy status; (2) Interventions (I): RCTs in which participants in one arm should use fixed-dose coformulated DHP; (3) Comparisons (C): the efficacy of DHP with ACT antimalarial(s), non-ACT antimalarial(s), or placebo, (4) Outcomes (O): the proportion of patients with parasitaemia and provided the effect estimates (or allowed data for computation of an effect estimate) relative risk (RR), hazards ratio (HR) or odds ratio (OR) and their corresponding 95% confidence interval (CI). If a selected study included more than one comparator, each comparison was regarded as a separate study. We included studies with participants having mixed infection (e.g. *P. vivax* and *P. falciparum*) for subgroup analysis. Studies on economic evaluation, mathematical modeling or pharmacokinetics were not included.

### Outcomes

In the present review, outcomes were defined as follows.

#### Primary outcomes

1) Polymerase chain reaction (PCR) confirmed parasitological failure by day 28 after starting treatment (defined as parasitaemia on any day between day 3 and day 28, irrespective of clinical condition);2) PCR-confirmed parasitological failure by day 42 after starting treatment (defined as parasitaemia on any day between day 3 and day 42, irrespective of clinical condition); 3) PCR-confirmed parasitological failure for more than 42 days after starting treatment (defined as parasitaemia on any day between day 3 and day 63, irrespective of clinical condition).

#### Secondary outcomes

1) Safety outcomes (incidence of adverse events); 2) Resolution of fever (i.e. time to fever clearance (FCT)) and 3) time to parasite clearance (PCT).

An adverse event (AE) was defined as any unfavorable, unintended sign, symptom, syndrome or disease that develops or worsens with the use of a medicinal product, regardless of whether it is related to the actual medicinal product. A serious AE was defined as any untoward medical occurrence that at any dose; resulted in death; was life threatening; requiring hospitalization or prolongation of hospitalization; resulted in a persistent or significant disability or incapacity; or caused a congenital anomaly or birth defect [23].

### Data extraction and quality assessment

Two investigators read all the titles and abstracts collected through the electronic search and filtered article(s) potentially eligible for the present study. The two investigators collected information on baseline characteristics of study design, participants, characteristics of the experimental drug, confirmation of *P. vivax* infection, duration of follow up, outcomes for each included articles, using a piloted data abstraction form. Power calculation for the required sample size was also assessed, if available. If articles contained information on the same or overlapping study population, we included the study with the most complete information.

The two investigators independently assessed risk for bias, following the procedures suggested by the Cochrane Risk of Bias tool [24]. The domains were random sequences generation, allocation concealment, blinding of outcome assessment and they are classified as ‘low risk’ ‘high risk’ or ‘unclear risk’. Discrepancies were resolved through consensus. All data were collected on an intention-to-treat (ITT) basis whenever possible. The two investigators also assessed the confidence in estimates of treatment effects according to the Grading of Recommendations Assessments, Development, and Evaluation (GRADE) approach [25] and made judgments on risk for bias, precision, consistency, and likelihood of publication bias. For precision, assessments were made on the basis of the boundaries of the CI of the summary estimates. Consistency was judged by visual inspection of forest plot for similar directions of effect from individual studies and for narrow ranges of effect size across studies. Publication bias was assessed by visual inspection of funnel plots.

### Data analyses

We performed meta-analysis when 2 or more individual studies were suitable for pooling on the basis of similarity. Parasitological efficacy was compared with the rate of parasitological failure between DHP and the comparator drug. The parasitological efficacy was pooled by HR and corresponding 95% CI, if studies reported time-to-event outcomes by the Kaplan-Meier method or data available for calculation of HR, using formula described by Parmer [26] and Tierney [27], as appropriate. Otherwise, we used the DerSimonian and Laird random effect model when pooling data and calculated RR and corresponding 95% CI. To test the robustness of our results, we reanalyzed the effect estimate using the data from per protocol analysis [35]. We also planned to reanalyze the effect estimates by excluding individual studies from the meta-analysis, if data permit.

We assessed heterogeneity by chi-square test and the *I* ^2^ test. *I* ^2^ value greater than 50% represented substantial heterogeneity [24]. Meta-analysis was done using RevMan Version 5·2 (The Cochrane Collaboration, Nordic Cochrane Centre, Copenhagen, Denmark) and MetaXL (www.epigear.com). We summarized the confidence in our findings by using GRADEProfiler version 3,6 [25].

The protocol of the present study is available in PROSPERO (CRD: CRD 42013004625) [28]. The methods and findings of the present review have been reported according to the preferred reporting items for systematic reviews and meta-analyses (PRISMA) [29] (Checklist S1).

## Results

**Figure 1:**
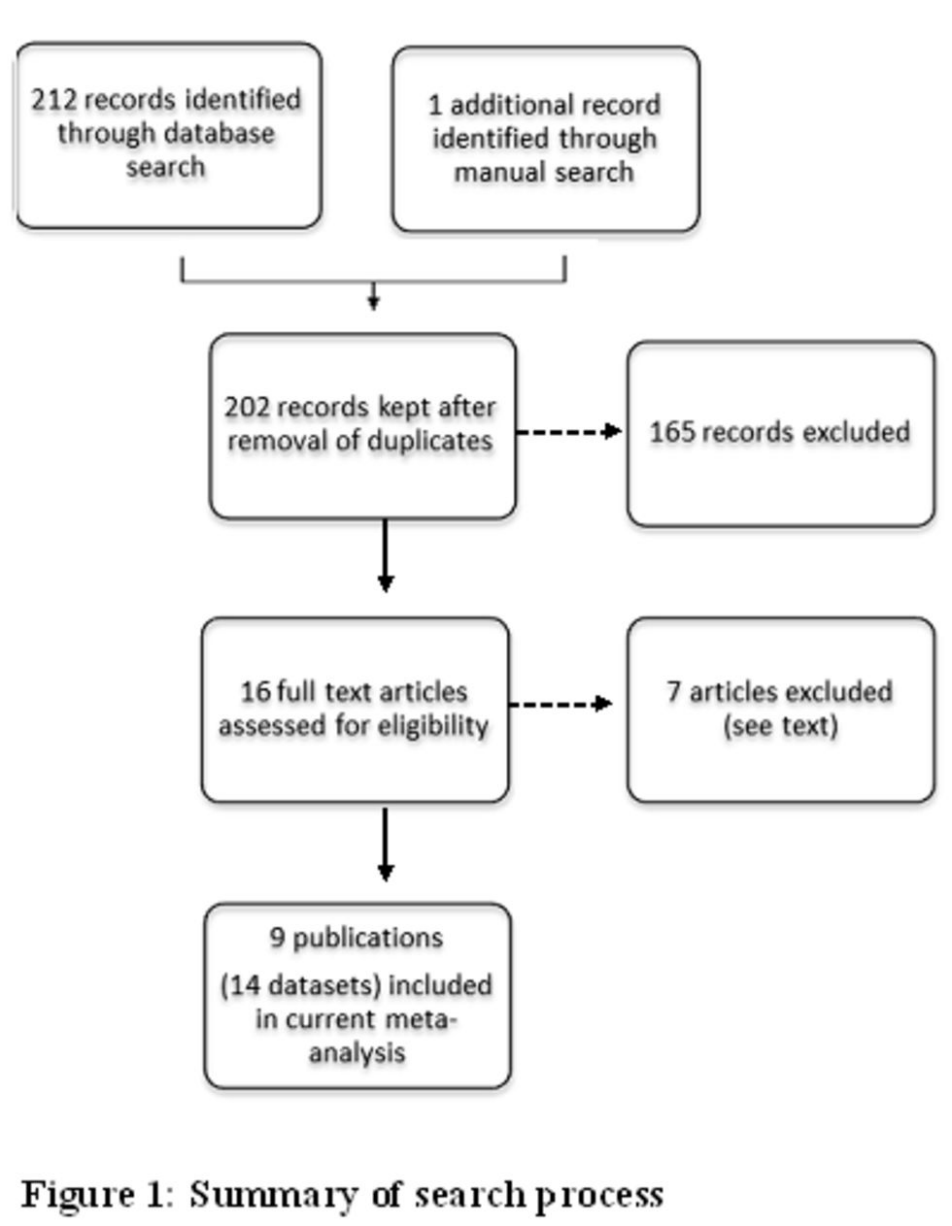
Summary of studies search.

Figure 1 provides the summary of search and selection of studies. A total of 213 records were identified, of which a final count of 16 studies were selected which met the inclusion criteria [30-45]. The meta-analysis of efficacy studies included 9 studies incorporating 14 datasets [30-38]. Amongst the studies initially retrieved, the Ashley study [30], provided data only for mixed infections. Other studies were excluded for a variety of reasons including: 1) no *P. vivax* infection detected at enrollment [39], 2) differentiating information on *P. vivax* and/or mixed infections was not provided [40,41], 3) single arm studies [42,43], 4) only pooled data was available [44] or 5), the study looked at intermittent preventive treatment (IPT) on a monthly basis [45].

Baseline characteristics of the included studies are presented in Table S1. The majority of studies (77%; 7/9) were performed in Southeast Asian countries. The remaining two were carried out in Papua New Guinea and Afghanistan, respectively. All included studies were recent publications ranging from years 2005-2013 and all were in English. Only one study was carried out with children under 5-year old [33]. The majority of participants in the primary studies were males, except a study in Afghanistan [31]. None of the included studies reported electrocardiogram (ECG) monitoring of participants during the period of their study.

In the present review, DHP was compared with artesunate-mefloquine (MAS3) in two trials [30,37], with artemether-lumefentrine (AL) in two trials [33,36], with artesunate-amodiaquine (AAQ) in one trial [33], with artemisinin-naphthoquine (AN) in one trial [38], with CQ plus sulfadoxine-pyrimethamine (CQ-SP) in one trial [33] and with CQ alone in two trials [31,35]. All studies assessed DHP, except a recently published RCT in Indonesia assessed DHP plus primaquine (PQ) vs AAQ plus PQ for radical treatment of vivax malaria [38]. Minimum effective plasma concentration of combined CQ-desethylchloroquine was done in two [33,35] of three studies where CQ was a comparator. Most of the included studies were judged to have a ‘low risk of bias’ on the basis of the random sequences of generation, adequate allocation concealment and blinding to the laboratory staff. Sample size calculations were done in 8 studies (88.9%) (Table 1).

**Table 1:** Risk of bias of the included studies.

**Study author**	**Ref.**	**Random sequence generation**	**Allocation concealment**	**Blinding of laboratory staff**	**Sample size calculation**
Ashley	[30]	yes	yes	yes	no
Awab	[31]	yes	yes	yes **^*a*^**	yes
Hasugian	[32]	yes	yes	no	yes
Karunajeewa	[33]	yes	unclear	yes	yes
Pasaribu**^*e*^**	[34]	yes	yes	NA **^*a*^**	yes
Phyo	[35]	yes	yes	no	yes
Ratclif	[36]	yes	no	yes **^*a*^**	yes
Smithuis	[37]	unclear	yes	yes **^*a*^**	yes
Tjitra	[38]	yes	yes	yes **^*a*^**	yes

Ref.: Reference number; Yes: low risk of bias; No: high risk of bias; unclear: unclear risk of bias;

*a* Open label; NA: detailed information not available

### 1: Effect of intervention stratified by comparator drugs

#### i): PCR-confirmed parasitaemia at days 28, 42 and at day >42-63

**Figure 2:**
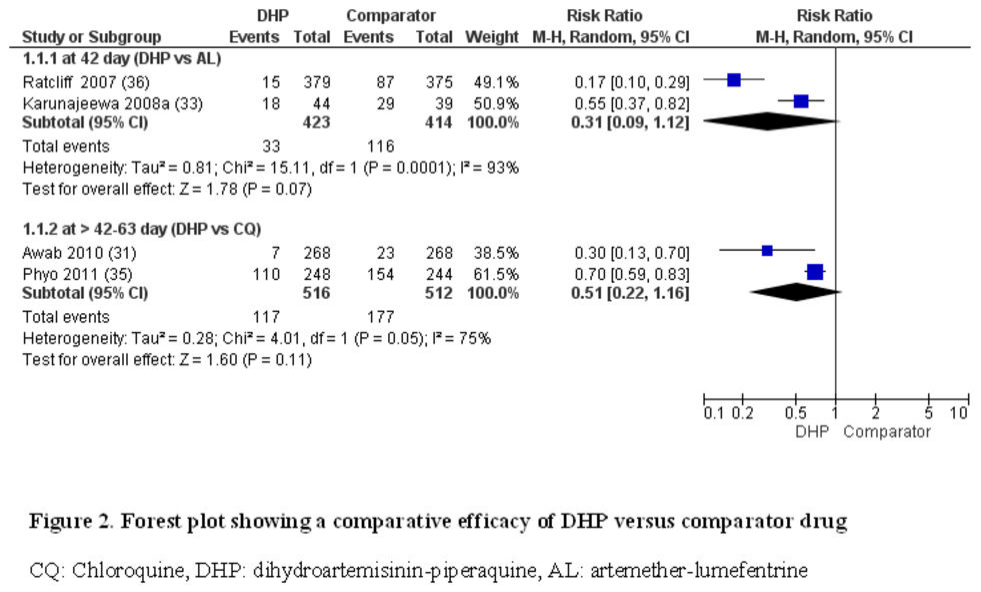
Forest plot showing a comparative efficacy of DHP versus comparator drug CQ: Chloroquine, DHP: dihydroartemisinin-piperaquine, AL: artemether-lumefentrine.

**Figure 3:**
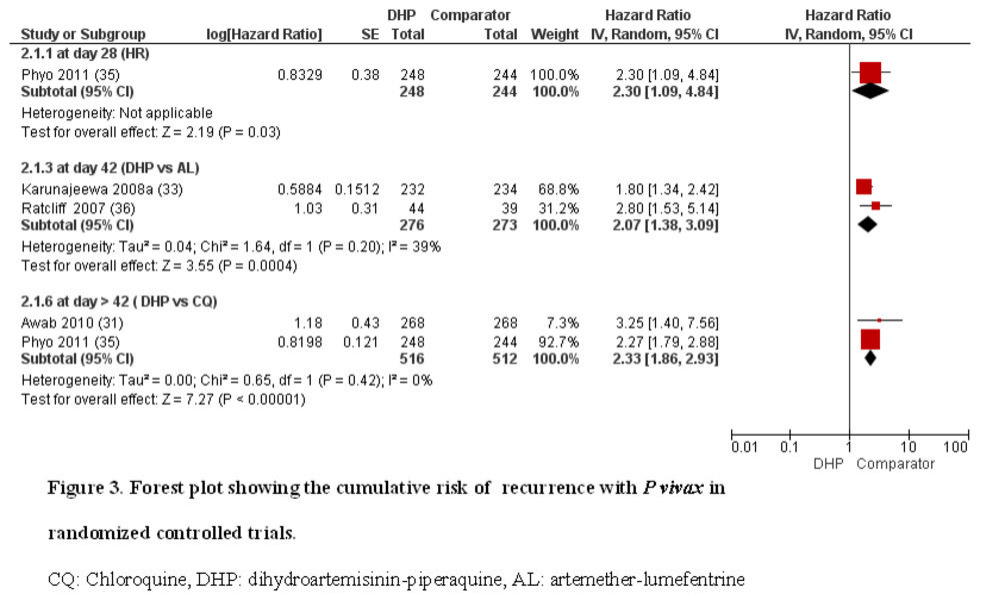
Forest plot showing the cumulative risk of recurrence with *P vivax* in randomized controlled trials. CQ: Chloroquine, DHP: dihydroartemisinin-piperaquine, AL: artemether-lumefentrine.

**DHP versus CQ:** At day > 42- 63, two studies (n = 1028) [31,35] showed comparable efficacy (22.7%, 117/516 vs 34.5%, 177/512; RR: 0.51, 95% CI: 0.22-1.16, *I* ^2^: 75%) in treating uncomplicated *P. vivax* malaria (Figure 2). As an alternative, we did the pooled analysis, using the fixed-effect model, DHP was better efficacy than CQ (RR: 0.63, 95% CI: 054-0.73, *I* ^2^: 74%). Of note is the substantial statistical heterogeneity. The results with HR indicated the higher cumulative risk of recurrence in the CQ group (HR 2.33, 95%CI 1.86, 2.93, *I* ^2^: 0%) (Figure 3).

**DHP versus AL:** At day 42, two studies (n = 837) [33,36] showed a comparable efficacy between DHP and AL (7.8%, 33/423 vs 28% 116/414, RR: 0.31, 95% CI 0.09-1.12, *I* ^2^: 93%). We reanalyzed the data, using the fixed-effect model and DHP showed better efficacy than AL (RR: 0.27, 95% CI: 0.19-0.36, *I* ^2^: 93%); due to substantial heterogeneity, it is not ideal for the pooled estimate (Figure 2). The results with HR showed the higher cumulative risk of recurrence in the AL group (HR 2.07, 95%CI 1.86, 3.09, *I* ^2^: 39%) (Figure 3).

#### ii): Subgroup analysis

We planned to stratify analyses by brand of DHP and age groups. Due to limitations in the data, we are unable to perform this analysis.

### 2: Fever and parasite clearance time in hours

Inadequate data restricted the ability to conduct a pooled analysis of FCT and PCT. An individual study [33] showed shorter mean PCT in the DHP group than that of CQ-SP (MD: 1.9, 95% CI: -2.77 to- 1.03), but a comparable mean PCT with AL (MD: 0.2, 95% CI: -0.77 to 0.31) or ART-SP (MD: 0.1, 95% CI: -0.55 to 0.75).

### 3: Adverse events

Due to difficulties in collecting the symptoms reported or exclusively relating to *P. vivax* infection, pooled estimates of AE incidences were not attainable. An individual study [35] showed that vomiting was less frequent in DHP compared to CQ (RR: 0.27, 95% CI: 0.11-0.66). DHP related SAE was not reported in any studies identified for the present review.

### 4: Mixed infections

As a subgroup analysis, two separate studies with mixed infection (with *P. falciparum*) showed a comparable efficacy between DHP and MAS3 [30] (RR: 1, 95% CI: 0.5-2.0) or AL [35] (RR: 0.83, 95% CI: 0.58-1.18).

### 5: Sensitivity analyses

For robustness of analysis, we reanalyzed the effect estimates, using the data from per protocol analysis [35]. At > 42-63 day, DHP and CQ also showed a comparable efficacy (RR 0.5, 95% CI, 0.22-1.15, *I* ^2^: 75%). Although we planned to reanalyze the effect estimates by excluding individual studies from the meta-analysis, this was not possible due to inadequate studies. According to the GRADE criteria to interpret results, further research is likely to have an important impact on our confidence in the estimate of effect and may change the estimate (Figure S1)**.**

## Discussion

The present study attempted to address the comparative efficacy and safety of DHP with respect to other antimalarial agents for the treatment of uncomplicated *P. vivax* malaria in patients living in endemic countries, mainly those in Southeast Asia. Clinical responses to treating malaria patients have an important role as a decision variable for use by policy makers.

### Efficacy

Based on the available data, our findings indicated that parasitological failure was lower in DHP than that of CQ or AL This could be explained by the fact that PPQ has the longest half- life of the drugs (compared to other partner drugs in ACT) and exposure to therapeutic drug levels over many parasite cycles is an important determinant of response [15,33]. The artemisinin component of DHP contributes significantly to the initial therapeutic response [35], but would not be expected to affect subsequent relapse or reinfection [31,35,38]. Studies documented that *P. vivax* parasite does not cause marked sequestration [11]. As all stages of asexual development are present in the peripheral blood, the initial decline in the level of parasitaemia following drug treatment of *P. vivax* malaria reflects antimalarial activity and not a combination of accelerated parasite clearance and sequestration [11,15]. This was supported by adequate plasma concentration of CQ in patients of the primary studies. Both CQ and DHP pose long elimination phases and therefore persistent (and adequate) blood concentrations, delaying the time to the first relapse. In areas where CQ sensitive parasites predominate, the prolonged post-treatment prophylaxis of CQ usually provides a minimum of 28 days without recurrence.

The relatively shorter mean PCT of DHP compared with CQ-SP would result in a relatively faster initial symptomatic response, increasing the confidence of parents/guardians of children as well as adult patients regarding the new drug. This is highly important from a clinical viewpoint. Moreover, the SP component of a drug is potentially important in settings where *P. vivax* is dominant and there is high prevalence of G6PD deficiency such as in certain ethnic groups. For example, Afghani and Asian patients can suffer significant haemolysis due to some antimalarials. The rapid development of resistance to SP when the drug is employed on the national or regional scale is attributable to the requirement [46].

Of note is that recurrences of *P. vivax* may consist of a mixture of relapses from dormant liver stage (hypnozoites), recrudescences of the erythrocytic infection (due to inadequate drug levels or resistance), and reinfections acquired from additional inoculations. It is not possible with current methodologies to distinguish reliably between these possibilities [31]. The interpretation of genotyping in the context of relapsing *P. vivax* infections is uncertain [47]. Therefore, genotyping of *P. vivax* in antimalarial drug trials has not been carried out as more than half of the parasites that caused the relapse had a different genotype from those that caused the primary infection [47,48]. A high rate of mixed-genotype infections occur even in settings where transmission is low [49], and true relapses caused by reactivated hypnozoites, cannot be ruled out or confirmed [47].

Initial parasite clearance was significantly faster after DHP in the present study and this was consistent with the pharmacodynamic properties of ACTs observed in *P. vivax* [11]. The rapid clearance of parasites in the CQ group, and the fact that failures were not seen before day 28 in an individual study [35] suggest that recrudescence associated with CQ resistance did not contribute significantly to the number of recurrences. For these reasons, the majority of recurrences observed in this study are more likely to be relapses [31]. As such, radical treatment of vivax malaria requires treatment with PQ. The relapse interval of *P. vivax* in Southeast Asians has traditionally been reported to be 6 weeks [50]. There are no genotyping methods that reliably distinguish relapses from new infections [47-49], and long-term cultures of *P. vivax* cannot be maintained to confine in vitro testing of drugs to assays on fresh isolates. Therefore, it is difficult to document unequivocal cases of treatment failure in areas where resistance is emerging, but any *P. vivax* infection that occurs within 28 days after the start of CQ treatment, whether recrudescence, relapse, or new infection, has grown through residual CQ concentrations in blood. If these concentrations are adequate, then, by definition, the infection is resistant [51]. Minimum effective plasma concentration of combined CQ-desethylchloroquine was assessed in some of the included studies, which further supported this claim. The relatively higher risk of parasitological failure in children under 5 years treated with DHP [35] was presumably attributed to both from lower immunity and lower blood PPQ concentrations in this group [39]. The latter was supported by a population pharmacokinetic study. Physiological processes do not scale linearly with body weight, and consequently children need a higher body weight-based dose than adults to achieve comparable drug concentration [52]. Therefore, weight adjusted higher doses may be required in children compared with adults in order to ensure adequate drug exposure [39].

### Mixed infection

Outside Africa, mixed infections of *P. falciparum* and *P. vivax* were common [31,36]. The effects of DHP in suppressing relapses of *P. vivax* infection in patients with mixed infections at baseline have merits. The delay in relapse and reinfections conferred by DHP gave patients a lengthened period without symptomatic malaria, allowing for a greater time for haematological recovery [32,36] and a subsequent reduced risk of anemia [32,36,38]. It also substantially reduced further transmission to the mosquito vector. Although such public-health implications of these benefits need to be confirmed by longer follow-up [36], it suggests potential programmes.

Of human malaria infections, *P. vivax* accounts for over half of all malaria transmitted outside Africa [2]. As almost all RCTs studies included in the present analysis were carried out in the Asian region, the current findings should be substantiated with the studies on efficacy of DHP for the treatment of vivax malaria in other endemic countries.

For highly effective treatments, it is more appropriate to show that a treatment is non-inferior or not worse than the standard treatment, i.e. that the difference in failure rate is not higher than a pre-specified non-inferiority margin [23]. Along this thread, published studies have documented that DHP is non-inferior than any existing antimalarial drugs in treating *P. falciparum* infection [18-20]. Due to the small number of studies, our findings could not prove that DHP is non-inferior to any existing antimalarial drugs in treating *P. vivax* infection. Future studies of well designed with adequate samples assessing efficacy of DHP in patients having *P. vivax* infection are recommended. Studies in endemic countries where *P. vivax* is proportionally dominant would be of great value.

Without radical treatment for *P. vivax* the numbers of patients who experience one or more, two or more, three or more relapses are exponential [53]. Additional work is required to establish how best to combine this treatment with appropriate antirelapse therapy (PQ or any drug under development), which is beyond the objective of the current study.

### Strengths and limitations of the study

Our review has strengths. Clinically important differences in the effect of treatment may be obscured if the proportions of survivors or recovered individuals in the treatment group are simply compared to that of the control group at a single point in time, such as at the conclusion of the trial. Time-to-event analysis is, therefore, a potentially more powerful and informative method of analysis [54,55]. The Kaplan-Meier method is preferred for statistical analysis of data on drug efficacy. The advantage of survival analysis is that it takes into account data on patients who were lost to follow-up or withdrawn from the study, in particular patients with reinfection [24]. The present review also showed evidence originated from the primary studies measured with HR, which is a merit. Furthermore, if not all, many studies included in the present review were of high quality.

Despite this, limitations also exist in the present study. Treatment failure attributable to ‘genuine resistance’ [56] was not confirmed with measurements of plasma concentrations of PPQ levels in all studies identified for this review. As the chemical instability of DHA is a concern [57], it is valuable to address efficacy of DHP according to pharmaceutical formulation used for the RCT. However, we are unable to stratify the analyses by brand of DHP. An unequal randomization was done in almost all included studies, which might give unbalanced bias of outcome assessment. On the other hand, such unequal randomization (e.g., 2:1) could provide more precise estimates of DHP cure rates and to provide more patients for the safety database of DHP [42]. Only a few studies compared with the same antimalarial drugs and reported the outcomes of the same follow-up duration, which created some difficulties in pooling of outcome data. The small number of studies with the small sample size in some the studies means that the possibility of type II statistical errors cannot be ruled out, as the selected studies were not powered to test for differences in the outcomes. The present work had some methodological difficulties with regard to pooling of results. For example, wide variations in data reporting made it difficult to compare the incidences of AE. A possible reason is that these drug-related symptoms could not be differentiated from malaria symptoms as they are transient and disappear 1-days after treatment [58]. Three deaths were reported in DHP group carried out in Thai Myanmar border and overall, these fatalities were considered as being unlikely to have resulted from its treatment with DHP [31]. The lack of application of ECG to measure possible cardiotoxicity in the included studies is a concern. Although more supporting safety data from RCTs would be reassuring [30], it is acknowledged that pharmacovigilance on this drug has not yet been extensive and some caution is still warranted. An experimental study suggests that DHP (and AL) neither display a significant potential proarrhythmic risk nor induce potential Torsade de pointes (TdP) [59]. We applied GRADE criteria to interpret results and concluded that further research is likely to have an important impact on our confidence in the estimate of effect and may change the estimate.

## Conclusion

Findings suggest that DHP is better than CQ and AL in the treatment of uncomplicated *P. vivax* malaria. Future RCTs with other ACTs are recommended. Additional work is required to establish how best to combine this treatment with appropriate antirelapse therapy (PQ or other drugs under development).

## Supporting Information

Checklist S1 **PRISMA checklist.**
(RTF)

Figure S1 **Summary evidence of the effect estimation on the basis of GRADE criteria.**
(TIF)

Table S1 **Baseline characteristics of the included studies.**
(RTF)
