# Supplementary figures and images for: Efficacy and Safety of Dihydroartemisinin-Piperaquine for Treatment of Plasmodium vivax Malaria in Endemic Countries: Meta-Analysis of Randomized Controlled Studies
Source: PLoS One. 2013 Dec 3;8(12):e78819. doi: 10.1371/journal.pone.0078819 (PMC3848966; doi:10.1371/journal.pone.0078819)

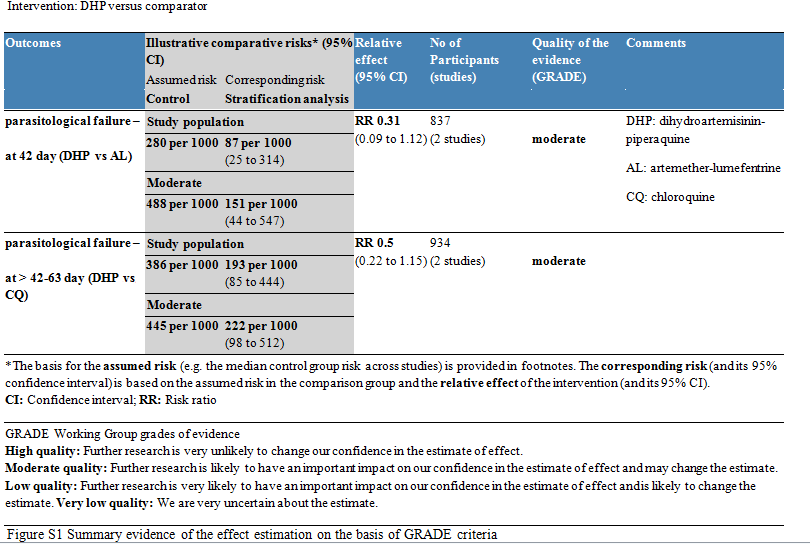

Supplement: Figure S1 — Summary evidence of the effect estimation on the basis of GRADE criteria. (TIF) [file pone.0078819.s002.tif]
