# Supplementary material for: Efficacy and Safety of Dihydroartemisinin-Piperaquine for Treatment of Plasmodium vivax Malaria in Endemic Countries: Meta-Analysis of Randomized Controlled Studies
Source: PLoS One. 2013 Dec 3;8(12):e78819. doi: 10.1371/journal.pone.0078819 (PMC3848966; doi:10.1371/journal.pone.0078819)
Supplement: Table S1 — Baseline characteristics of the included studies. (RTF) [file pone.0078819.s003.rtf]

Table S1.  Baseline characteristics of included studies in the meta-analysis

Study author	Ref.	Study country	Malaria transmission	Comparator	Sample size 
(DHP/ comparator)	Age a	Male  	Brand 	Supervised dose	
Ashley	30	Thialnd	unstable, low, seasonal	MAS3	16/16 b	10 (3-14) e	30% c	Artekin,
(Holleykin, PRC)	yes	
Awab	31	Afghanistan	low/high	CQ	268/268	>14 :
41%	41% 	Artekin,
(Holleypharm, PRC	yes	
Hasugian 	32	Indonesia	unstable	AAQ	54/60	>14 : 55%	59% c	  Artekin (Holleykin, PRC)	yes	
Karunajeewa	33	Puapa New Guina	hyper endemic	CQ-SP
AL
ART-SP	44/61 (CQ-SP)
44/39 (AL)
44/51 (ART-SP)	37 (6-60) mt	NA	DHP (Holley- Cotec,  PRC)	yes	
Pasaribue	34	Indonesia	unstable	AAQ+PQ	164/167	14.5 (2-70)	51.8%	Arterakine (Pharbaco Central)	yes	
Phyo	35	Thailand	unstable, low, seasonal	CQ	230/207	18 (1-57) 	70% c	Duocotexin (Holley, PRC)	yes	
Ratclif	36	Indonesia	unstable	AL	90/85	>14 : 56% c	58% c	Artekin,
(Holleykin, PRC)	yes	
Smithuis	37	Myanmar	unstable, low, sesonal	MAS3-F
MAS3-L	15/28 c
15/11 b	>14 : 11%c	69%c	NA	yes	
Tjitra	38	Indonesia	unstable	  AN	72/76	26.2 ±9.2	85% c	Duo-Cotecxin,
(Holle-Cotec, PRC	yes d	

amedian age in years and range, unless otherwise stated; b: mixed-infection; c: based on total participants (Pf, Pv & mixed);  
d: hospitalized patients; e: DHP + PQ
Ref: reference number;  mt: month; brand: brand of DHP; Pharbaco Central: Pharbaco Central Pharmaceuticals, Vietnam; 
PRC: People's Republic of China
AAQ: Amodiaquine plus artesunate; DHP: dihydroartemisinin-piperaquine; PQ: primiquine for 14 days; MAS3-F: Artesunate–mefloquine (fixed) ; MAS3-L: Artesunate–mefloquine (loose tablets). 
